# Can Some Marine-Derived Fungal Metabolites Become Actual Anticancer Agents?

**DOI:** 10.3390/md13063950

**Published:** 2015-06-19

**Authors:** Nelson G. M. Gomes, Florence Lefranc, Anake Kijjoa, Robert Kiss

**Affiliations:** 1 ICBAS—Instituto de Ciências Biomédicas Abel Salazar, Rua de Jorge Viterbo Ferreira 228, 4050-313 Porto, Portugal; E-Mails: goncalomortagua@hotmail.com (N.G.M.G.); ankijjoa@icbas.up.pt (A.K.); 2 Interdisciplinary Centre of Marine and Environmental Research (CIIMAR), Universidade do Porto, Rua dos Bragas 289, 4050-123 Porto, Portugal; 3 Service de Neurochirurgie, Hôpital Erasme, Université Libre de Bruxelles, 808 Route de Lennik, 1070 Brussels, Belgium; E-Mail: florence.lefranc@erasme.ulb.ac.be; 4 Laboratoire de Cancérologie et de Toxicologie Expérimentale, Faculté de Pharmacie, Université Libre de Bruxelles, Campus de la Plaine, CP205/1, Boulevard du Triomphe, 1050 Brussels, Belgium

**Keywords:** marine fungi, natural products, anticancer, chemotherapeutic, cytotoxic, multidrug resistance, *in vivo* antitumor, non-apoptotic, pro-apoptotic, preclinical

## Abstract

Marine fungi are known to produce structurally unique secondary metabolites, and more than 1000 marine fungal-derived metabolites have already been reported. Despite the absence of marine fungal-derived metabolites in the current clinical pipeline, dozens of them have been classified as potential chemotherapy candidates because of their anticancer activity. Over the last decade, several comprehensive reviews have covered the potential anticancer activity of marine fungal-derived metabolites. However, these reviews consider the term “*cytotoxicity*” to be synonymous with “*anticancer agent*”, which is not actually true. Indeed, a cytotoxic compound is by definition a poisonous compound. To become a potential anticancer agent, a cytotoxic compound must at least display (i) selectivity between normal and cancer cells (ii) activity against multidrug-resistant (MDR) cancer cells; and (iii) a preferentially non-apoptotic cell death mechanism, as it is now well known that a high proportion of cancer cells that resist chemotherapy are in fact apoptosis-resistant cancer cells against which pro-apoptotic drugs have more than limited efficacy. The present review thus focuses on the cytotoxic marine fungal-derived metabolites whose ability to kill cancer cells has been reported in the literature. Particular attention is paid to the compounds that kill cancer cells through non-apoptotic cell death mechanisms.

## 1. Introduction

### 1.1. Cancer Epidemiology

According to *World Cancer Report 2014*, which was published by the World Health Organization’s International Agency for Research on Cancer, the global incidence of cancer rose to an estimated 14 million new cases in 2012, and this figure is expected to rise to an annual 19.3 million cases by 2025 [1]. The same report also states that, among the commonest cancers, lung cancer was found to be the highest, making up 13% of the total number in 2012, followed by breast cancer (11.9%), colorectal cancer (9.7%), and prostate cancer (7.9%). In addition, most cancers occur in the less developed regions of the world, with 60% of cancers and 70% of cancer deaths occurring in Africa, Asia, and Central and South America.

Torre *et al.* [2] argued that the occurrence of cancer is increasing because of the growth and aging of the population, as well as the increasing prevalence of established risk factors such as smoking, overweight, physical inactivity, and changing reproductive patterns associated with urbanization and economic development. The same authors have also reported that the other leading causes of cancer deaths in more developed countries included colorectal cancer among males and females and prostate cancer among males, whereas liver and stomach cancer among males and cervical cancer among females were the leading causes of cancer deaths in less developed countries [2].

### 1.2. The Role of Natural Products in Cancer Therapy

The role of natural products in drug discovery is tremendous, specifically for the development of chemotherapeutic agents, and these products are the primary contributing source that feeds the current anticancer clinical pipeline. The contribution of natural sources is not only limited to the direct application of unmodified secondary metabolites but also extends to their derivatives such as semi-synthetic analogs of lead structures, as well as to synthetic structural mimics inspired by natural products. A recent survey by Newman and Giddings [3] was used to analyze the sources of the 191 chemotherapeutic agents that were marketed from the late 1930s to the end of 2012, and this survey indicates that 89 can be ascribed to natural products or their modified forms, and 39 correspond to synthetic compounds with a natural origin, giving a total of only 63 (33%) anticancer agents that are classified as truly synthetic in origin.

The large number of plant-derived anticancer drugs that are currently available clearly supports the leading role of terrestrial flora in cancer drug discovery, including several chemotherapeutic agents such as the blockbuster drug paclitaxel (Taxol^®^), which was originally isolated from the Pacific yew tree *Taxus brevifolia*, as well as its semi-synthetic analogs docetaxel (Taxotere^®^) and the third generation taxane cabazitaxel (Jevtana^®^) [4,5]. Another successful group of plant-based antitumor drugs is the vinca alkaloids, namely vincristine and vinblastine, which are derived from *Catharanthus roseus* [6], the semi-synthetic camptothecin analogs irinotecan and topotecan [7,8], as well as the topoisomerase II inhibitors etoposide and teniposide, which are semi-synthetic derivatives of epipodophyllotoxin that was originally isolated from *Podophyllum peltatum* [9,10]. Despite their preponderant role in antibiotherapy, bacteria have also widely contributed to some of the most clinically useful drugs in the currently available chemotherapeutic arsenal. Several *Streptomyces*-derived active metabolites served as lead structures for the development of the most effective anticancer drugs such as the anthracyclines doxorubicin and daunomycin, as well as the mitosanes and bleomycin-related agents [11].

There is no doubt that the major class of natural product-based anticancer agents are derived primarily from lead compounds from the terrestrial environment, which was related to ethno-medical history as well as relatively easy accessibility to natural sources. However, the recognized potential of the marine environment as a potential resource for pharmacologically active secondary metabolites has led to the extensive chemical investigation of a myriad of marine organisms in recent decades, which has resulted in the emergence of an increasing number of candidates for the development of new cancer therapy drugs [12,13,14,15].

Despite the surprisingly vast number of new, structurally diverse, biologically active, marine-derived natural products, only four anticancer drugs have been developed into drugs that have been clinically approved by the FDA (Food and Drug Administration) or EMA (European Medicines Agency) so far. After receiving its FDA approval in 1969, the DNA polymerase inhibitor cytarabine was the first marine-derived drug that became available for clinical use. This chemotherapeutic agent is a synthetic analog of the nucleosides spongothymidine and spongouridine, which were extracted from the sponge *Cryptotethya crypta* in the late 1940s [16]. At present, cytarabine (Cytosar-U^®^) is primarily used as a single agent or in combination with mitoxantrone and daunorubicin to treat acute myeloid leukemia and for non-Hodgkin’s lymphoma and meningeal leukemia (DepoCyte^®^) [17]. Later, trabectedin, which is also known as ecteinascidin-743 (ET-743), a tetrahydroisoquinoline alkaloid originally isolated from the Caribbean tunicate *Ecteinascidia turbinata* [18,19], became clinically available in the EU and South Korea under the trade name Yondelis^®^ to treat soft tissue sarcoma and relapsed platinum-sensitive ovarian cancer [20]. It is worth mentioning that trabectedin is now commercially produced by hemisynthesis from the bacterial fermentation product cyanosafracin B [21]. The third marine-derived chemotherapeutic agent to receive FDA and EMA approval was the tubulin inhibitor eribulin mesylate (Halaven^®^), a synthetic derivative based on the structure of the macrocyclic polyether halichondrin B, which was isolated in 1986 from the sponge *Halichondria okadai* [22]. Halaven^®^ is currently used in the US, the EU and Asia to treat refractory metastatic breast cancer [23], and several other halichondrin B derivatives, including eribulin mesylate itself, are currently undergoing several clinical trials (Phase I to Phase IV) against other types of cancer [3]. Brentuximab vedotin is an immunoconjugate based on the fully synthetic derivative monomethyl auristatin E, and it was the most recent successful marine-derived anticancer drug to receive marketing authorization. With approval from the FDA in 2011 and the EMA in 2012, the monoclonal antibody monomethyl auristatin E (Adcentris^®^) has been used to treat Hodgkin’s and systemic anaplastic large cell lymphoma [24]. Dolastatin 10, the lead structure of monomethyl auristatin E, was originally reported as coming from the Indian Ocean sea hare *Dolabella auricularia* [25], but further studies revealed that its metabolic sources were the cyanobacteria of the genera *Simploca* and *Lyngbya* [26,27].

Although marine-derived drugs are under-represented in the current chemotherapeutic clinical arsenal, a vast number of candidates is undergoing preclinical and advanced clinical development, accounting for more than 1000 clinical trials listed in both NIH (National Institutes of Health) and European databases [14,28]. In reference to new lead structures, new applications against other cancer types for the currently marketed drugs, new synthetic derivatives, as well as new regimens in combination with other chemotherapeutic agents, these candidates will certainly provide new clinically useful agents for cancer treatment in the near future.

## 2. Marine Fungal-Derived Metabolites *versus* Cancer

Since the development of β-lactam antibiotics, fungi have been shown to be an extremely useful source of lead compounds for the development of new pharmaceuticals. Further examples of fungal-derived therapeutic agents include top-selling drugs such as the lipid-lowering agents atorvastatin and simvastatin, as well as the immunosuppressive agent cyclosporine A and the antifungal griseofulvin, among others [29,30].

Fungi from terrestrial sources have provided several lead structures for the development of many relevant drugs that are currently used in human medicine and continue to be extensively studied. In the last 25 years, several research groups from pharmaceutical companies and academic institutions have been paying attention to marine fungi as a source of new drug leads. The recent and systematic chemical characterization of fungi from the marine environment has provided a total number that currently exceeds 1000 new natural products [31]. Although most of these metabolites are closely related to those isolated from their terrestrial counterparts, several examples with unusual carbon skeletons and substitution patterns indicate that the marine strains may possess distinct biosynthetic capabilities [31,32,33]. Despite the emergence of an impressive number of new marine fungal-derived metabolites with promising pharmacological activities, only the broad-spectrum antibiotic cephalosporin C can be tracked back as a marine fungal-derived drug so far. Cephalosporin C was discovered from a fungus in a sample collected from the Sardinian coast, and the fungus was later identified as *Acremonium chrysogenum* [34]. The minor contribution of fungi in marine habitats as a source of drug leads for the current available therapeutic arsenal is likely related to the fact that the chemical investigation of these organisms as producers of pharmacologically relevant metabolites was long neglected until the end of the 1980s, with only 15 secondary metabolites produced by marine-derived fungi described until 1992 [32].

An increasing focus on the chemical and biological potential of marine fungi and on screening for new lead structures with promising anticancer activities led to the identification of several relevant compounds. Over the last few years, several metabolites produced by marine fungi have displayed potent anticancer effects, which were mediated by several mechanisms such as blocking key enzymes, stimulating death pathways or promoting growth arrest [35].

The current review, which covers the period from 1983 to 2014, emphasizes the direct effects of marine fungi-derived metabolites on the death of cancer cells. In other words, we did not review marine-derived fungal metabolites merely classified as compounds that displayed potential anti-angiogenic effects or immunologic effects.

### 2.1. Pro-Apoptotic Metabolites

It must be emphasized that most cytotoxic cell insults will lead to apoptosis, whether the cells are normal or cancerous. Thus, it is mandatory to distinguish between the direct and indirect pro-apoptotic effects of a given compound. As explained in Sub-Section 4.1, “*Are Pro-Apoptotic Compounds still Valuable Weapons to Combat Cancer?*”, cancers that resist chemotherapy will in fact resist direct pro-apoptotic insults because they contain a majority of apoptosis-resistant cells as in the case of metastatic cancers [36,37,38,39,40].

It is worth bearing in mind that the tools available to biologists and pharmacologists studying the mechanisms of action of the compounds that were characterized in the late 1990s or early 2000s were not as sophisticated as those available today. Thus, it is still possible that some compounds that were described as pro-apoptotic can also display other mechanisms of action [41,42]. We will revisit this concept in Section 4, “*How Could We Increase the Rate of Discovery of Marine-Derived Fungal Metabolites as Potential Anticancer Agents, at Least in Vitro?*”

**Figure 1:**
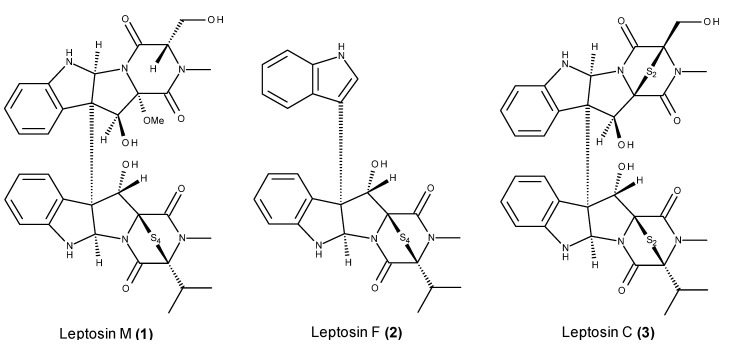
Structure of leptosin M (**1**), F (**2**) and C (**3**).

The leptosins are one of the largest classes of cytotoxic fungal metabolites and so far include 23 members, which correspond to a series of both monomeric and dimeric epipolythiodiketopiperazine alkaloids. These alkaloids were produced by the strain *Leptosphaeria* sp. OUPS-4, which was isolated from the brown alga *Sargassum tortile* collected from Tanabe Bay, Japan. All the leptosins, especially the dimeric members, showed cytotoxicity against the murine P388 lymphocytic cell line [43,44,45,46,47,48]. Leptosins M (**1**), F (**2**) and C (**3**) (Figure 1) revealed further anticancer effects. Leptosin M (1) displayed significant cytotoxicity against human cancer cell lines through the inhibition of two protein kinases, namely PTK and CaMKIII, and human topoisomerase II [47], and leptosins F (**2**) and C (**3**) are catalytic inhibitors of topoisomerase I [49]. Leptosin C (**3**) induced apoptosis and inhibited the Akt pathway [49]. The inhibition of apoptosis leads to the activation of cell survival factors (such as Akt), which in turn causes continuous cell proliferation in cancer tissues [50].

**Figure 2:**
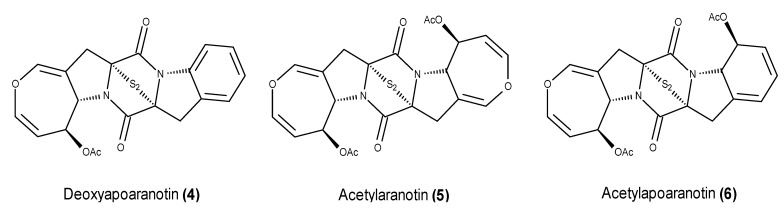
Structures of the diketopiperazine disulfides **4**–**6**.

A bioassay-guided fractionation of an EtOAc extract from the culture of the marine fungus *Aspergillus versicolor* KMD 901, which was isolated from a deep sea sediment collected from the Ulleung Basin in the East Sea of Korea, yielded a new diketopiperazine disulfide called deoxyapoaranotin (**4**), together with acetylaranotin (**5**) and acetylapoaranotin (**6**) (Figure 2) [51].

Compounds **4**–**6** were found to induce apoptosis in the HCT116 colon cancer cell line through both extrinsic and intrinsic pathways. In addition to the down-regulation of the anti-apoptotic proteins Bcl-2 and Bcl-xL and the up-regulation of the pro-apoptotic protein Bax, they were also found to cleave caspases-3, -9 and -8, suggesting that the apoptotic process is initiated by caspase 8. In addition to the *in vitro* apoptotic effect, acetylapoaranotin (**6**), which exhibited the most potent proliferation inhibition effects in HCT116, AGS, A549 and MCF-7 cancer cell lines, also induced a significant inhibition of tumor growth in an *in vivo* xenograft model [51].

**Figure 3:**
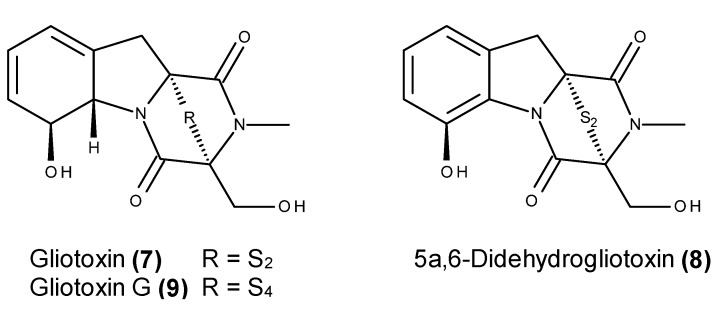
Structures of gliotoxin (**7**) and gliotoxin derivatives **8**–**9**.

Gliotoxin (**7**) (Figure 3), the first member of the epipolythiodiketopiperazine class and bridged diketopiperazine alkaloids, has been widely reported as constituent of several marine-derived fungi [52,53,54]. Recently, Nguyen *et al.* [55] reported the isolation and evaluation of the anticancer activity of gliotoxin (**7**) from *Aspergillus* sp. strain YL-06, which was isolated from a marine brown alga collected in Ulsan, Korea. This compound is pro-apoptotic in human HeLa cervix carcinoma and SW1353 chondrosarcoma cells through the activation of caspase-3, caspase-8 and caspase-9, the down-regulation of Bcl-2, the up-regulation of Bax and the release of cytochrome c (cyt c) [55].

Gliotoxin (**7**) was tested for its inhibitory activity against the cytosolic metalloenzyme FTase (farnesyltransferase) and the related prenyltransferase GGTase I (geranylgeranyltransferase I), which catalyze the prenylation of several cellular proteins that are involved in tumor progression such as the Ras family [56]. This compound displayed a dual inhibition of FTase and GGTase I-inhibiting protein isoprenylation and cell proliferation in breast cancer MCF-7 and MDA-MB-231 cell lines [57]. A chemical investigation of the fungus *Penicillium* sp. strain JMF034, which was isolated from deep sea sediments collected from Suruga Bay in Japan, resulted in the isolation of several gliotoxin-derived compounds. Gliotoxin (**7**) and its derivatives 5a,6-didehydrogliotoxin (**8**) and gliotoxin G (**9**) (Figure 3) exhibited potent inhibitory activity against the methylation regulation enzymes HMT G9a and HMT Set7/9 [53], which are commonly involved in the malignant transformation of cells [58]. The absence of activity from the other gliotoxin derivatives supported the key role of the disulfide and tetrasulfide bonds in the enzymatic inhibitory activity [53].

**Figure 4:**
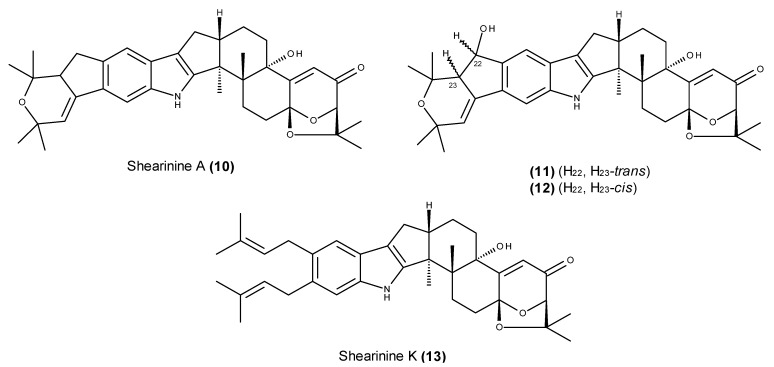
Structure of shearinine A (**10**) and its derivatives (**11**–**13**).

Several new derivatives of shearinine A (**10**) (Figure 4), a class of janthitrem-type indole terpenes, were reported from the endophytic fungus *Penicillium* sp. strain HKI0459, which was isolated from a mangrove plant collected near Xianen City, China [59]. At the same time, Smetanina *et al.* [60] reported the isolation of two additional shearinine A-related metabolites (**11**, **12**) (Figure 4) from the EtOAc extract of the fungus *Penicillium janthinellum*, which was collected from bottom sediments in Amursky Bay, Russia.

The new shearinine derivatives (**11** and **12**) were incorrectly named shearinine D and E [61], respectively, which were incorrect because these names had already been used by Xu *et al.* in their previous report [59] to describe other shearinine derivatives. Additionally, shearinine K (**13**) (Figure 4) was wrongly reported as a new compound called shearinine F by Smetanina *et al.*; it was incorrectly reported because it had already been isolated [59,61]. Although shearinine A (**10**) and its derivatives **11** and **12** induced apoptosis in human leukemia HL-60 cells, compound **12** also inhibited the EGF-induced malignant transformation of JBC-P^+^-CI41 cells in a soft agar model [60].

**Figure 5:**
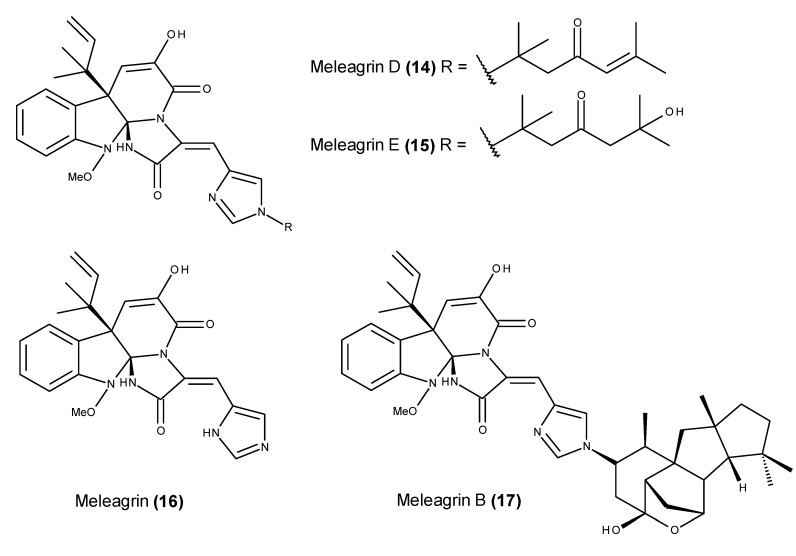
Structure of meleagrin (**16**) and meleagrin B (**17**), D (**14**) and E (**15**).

The culture of the marine fungal strain *Penicillium* sp. F23-2, which was obtained from a deep ocean sediment sample, yielded two new meleagrin analogs, namely meleagrin D (**14**) and E (**15**) (Figure 5) [62], in addition to the previously reported meleagrin (**16**) and meleagrin B (**17**) (Figure 5) [63].

Although the two new meleagrin analogs **14** and **15** displayed only modest cytotoxic activity against the A-549 cell line, meleagrin B (**17**) induced HL-60 cell apoptosis, and meleagrin (**16**) arrested the cell cycle through the G_2_/M phase [62]. Meleagrin (**16**) was later reported from other marine-derived *Penicillium* spp., and it exhibited relevant cytotoxic activity against several cancer cell lines [64,65].

**Figure 6:**
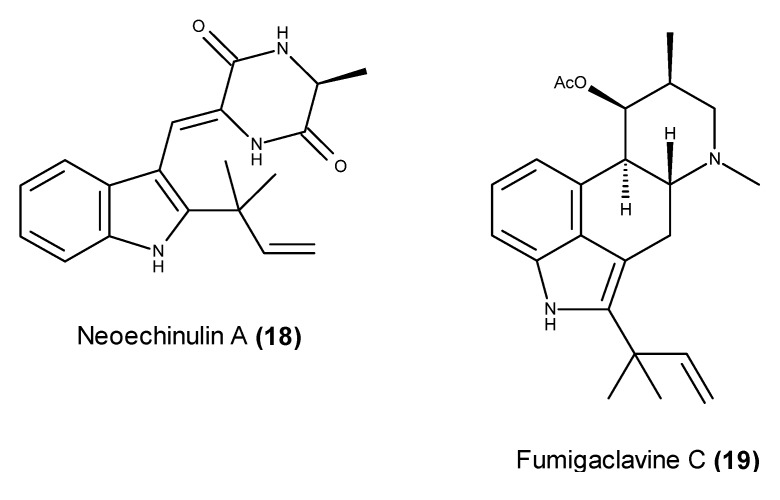
Structure of neoechinulin A (**18**) and fumigaclavine C (**19**).

Neoechinulin A (**18**) (Figure 6), a prenylated diketopiperazine alkaloid commonly reported from marine-derived *Eurotium* fungal strains [66,67,68], was isolated from the fungus *Microsporum* sp. MFS-YL, obtained from the surface of a red alga, which was collected at Guryongpo, PoHang in the Republic of Korea [69]. Neoechinulin A (**18**) displayed cytotoxic effects against human cervical carcinoma HeLa cells, and its induction of apoptosis in HeLa cells occurred through the down-regulation of Bcl-2 expression, the up-regulation of Bax expression, and the activation of the caspase-3 pathway [69].

A marine-derived strain of *Aspergillus fumigatus*, isolated from the surface of an unidentified alga, which was collected at Seosaeng-myeon in the Republic of Korea, was found to produce the indole alkaloid fumigaclavine C (**19**) (Figure 6) [70]. Fumigaclavine C (**19**) displayed pro-apoptotic effects in human MCF-7 breast cancer cells as well as the inhibition of MMP-2 and MMP-9 protease activity in these MCF-7 cells [70]. Fumigaclavine C (**19**) also down-regulated the NF-κB cell survival pathway [70].

**Figure 7:**
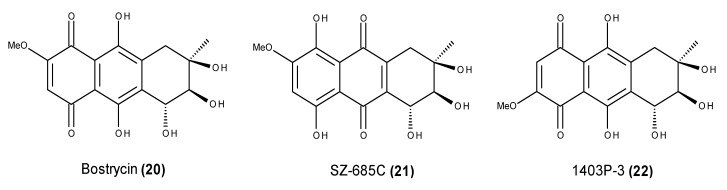
Structure of bostrycin (**20**), SZ-685C (**21**) and 1403P-3 (**22**).

The mangrove endophytic fungus *Nigrospora* sp. No. 1403, which was collected from the South China Sea, was found to produce three anthracenediones with interesting anticancer properties, namely bostrycin (**20**), SZ-685C (**21**) and 1403P-3 (**22**) (Figure 7) [71]. Bostrycin (**20**) and SZ-685C (**21**) are tautomers, and 1403P-3 (**22**) is a structural isomer, differing only in the position of the methoxyl substituent. Interestingly, bostrycin (**20**) was also reported in other marine-derived fungal species belonging to distinct genera such as *Aspergillus*, *Fusarium* and *Xylaria* [72,73,74].

Bostrycin (**20**) induces apoptosis in A549 non-small cell lung cancer (NSCLC) cells [75]. The bostrycin-induced pro-apoptotic effects in A549 NSCLC cells could be related to the up-regulation of microRNA-638 and microRNA-923 and the down-regulation of the PI3K/AKT protein pathway [75]. Bostrycin-induced cell death was promoted in the YCA1 null yeast strain but was partially rescued in the AIF1 null mutant in both fermentative and respiratory media, strongly indicating that bostrycin (**20**) induces apoptosis in yeast cells through a mitochondria-mediated but caspase-independent pathway [76]. A series of bostrycin derivatives was synthesized, and their cytotoxic activities were evaluated against MCF-7, MDA-MB-435, A549, HepG2 and HCT-116 cancer cells, with several compounds showing comparable cytotoxic activity to that of epirubicin [77]. However, these compounds also exhibited cytotoxicity towards normal MCF-10A breast cells [77].

SZ-685C (**21**), a bostrycin tautomer, induces pro-apoptotic effects in various cancer cell lines through the Akt/FOXO pathway [78]. SZ-685C (**21**) exhibited a direct apoptosis-inducing effect through both the extrinsic and intrinsic apoptotic pathways, and the phosphorylation of Akt and its downstream effectors, which are forkhead box protein O1 and forkhead box protein O3a, were down-regulated in SZ-685C-treated cancer cells [78]. Furthermore, the pro-apoptotic protein Bim was up-regulated by SZ-685C treatment, which is consistent with FOXO dephosphorylation [78]. Wang *et al.* [79] have noted that radioresistance is a major obstacle to the treatment of human nasopharyngeal carcinoma (NPC), and emerging evidence has demonstrated that miRNAs are involved in cancer therapy resistance. The miR-205-PTEN-Akt pathway is the key cell signaling pathway, and it is activated in the CNE2R cells upon SZ-685C treatment [79]. However, the Stat3-Jab1-p27 pathway might participate in the pro-apoptotic effect on CNE2 cells but not on CNE2R cells [79]. SZ-685C (**21**) exhibited pro-apoptotic activity in both radiosensitive and radioresistant NPC cells [79]. Although the mechanisms for the two cell lines were not identical, the pro-apoptotic effects were similar in both cell lines [79]. Additionally, SZ-685C (**21**) suppressed the Akt pathway and induced apoptosis in MCF-7/adriamycin (ADR) and MCF-7/Akt breast cancer cells that are resistant to ADR treatment, leading to antitumor effects both *in vitro* and *in vivo* [80].

Similarly, 1403P‑3 (**22**) also induced apoptosis in human breast cancer cells by blocking Akt activation [81]. 1403P-3 (**22**) showed potent cytotoxicity not only in drug-sensitive human epidermoid carcinoma parental KB cells but also in MDR KBv200 cells [82]. The apoptosis-inducing effect of this compound on KB and MDR KBv200 cells is mediated through the mitochondrial and death receptor pathways, with the mitochondrial pathway being independent of ROS (reactive oxygen species) and the activation of caspase-8 [82].

**Figure 8:**
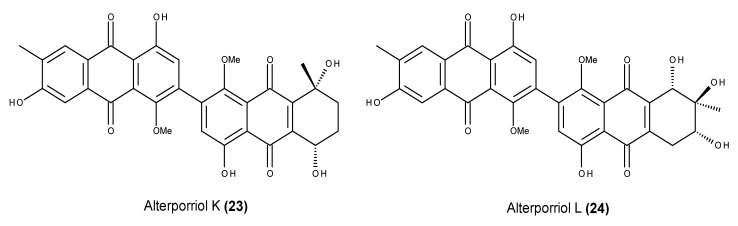
Structure of alterporriol K (**23**) and L (**24**).

Dimeric anthraquinones known as alterporriols K (**23**) and L (**24**) (Figure 8) were reported from the endophytic fungus *Alternaria* sp. ZJ9-6N, which was isolated from the fruit of the marine mangrove *Aegiceras corniculatum* in Zhanjiang, Guangdong province, China [83]. Both **23** and **24** displayed modest cytotoxic activity against two human breast cancer cell lines (MDA-MB-435 and MCF-7) [83]. Further studies were performed to elucidate the molecular mechanisms underlying the alterporriol L (**24**) cytotoxic effect, showing that induced apoptosis and necrosis. As suggested by the increased levels of cytosolic free calcium and ROS production as well as the induced loss of MMPo in alterporriol L-treated MCF-7 cells, the apoptotic and necrotic effects are supposedly derived from the destruction of mitochondrial functions [84].

**Figure 9:**
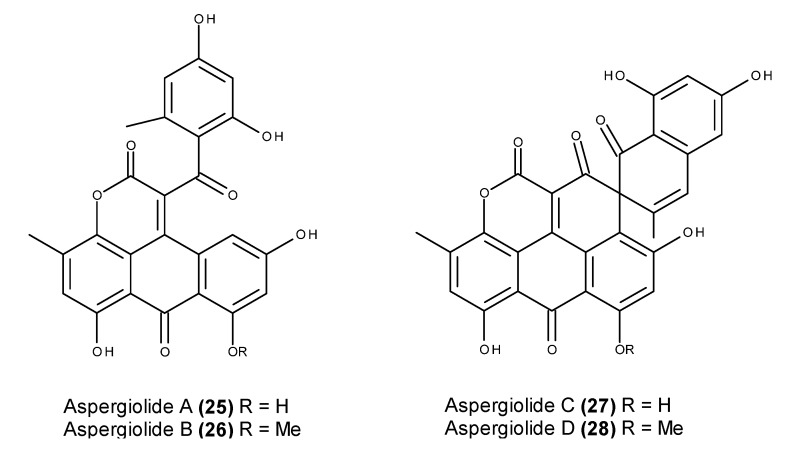
Structure of aspergiolide A (**25**), B (**26**), C (**27**) and D (**28**).

Aspergiolides A–D (**25**–**28**) (Figure 9) are cytotoxic anthraquinone derivatives with a naphtho [1,2,3-*de*]chromene-2,7-dione skeleton, and they were isolated from an EtOAc extract of cultures of the fungus *Aspergillus glaucus* HB1-19, which was obtained from a marine sediment collected in Fujian province, China [85,86,87,88].

Aspergiolide A (**25**) displayed an inhibitory activity against topoisomerase II that is comparable to that of adriamycin. This compound also induced apoptosis in hepatocellular carcinoma BEL-7402 cells via a caspase-dependent pathway [89]. Aspergiolides C (**27**) and D (**28**) were tested against a panel of 14 tyrosine kinases, revealing selective inhibitory kinase activities that displayed potent inhibitory activity in c-Met and the modest inhibition of RON and c-Src [88]. The selective inhibition of c-Met is apparently derived from the novel *spiro* [5.5.] undecane scaffold that binds to a specific site of the c-Met catalytic domain [88]. Furthermore, both enantiomers (**27** and **28**) significantly inhibited HGF-promoted MDCK cell migration by targeting the HGF/c-Met pathway [88].

**Figure 10:**
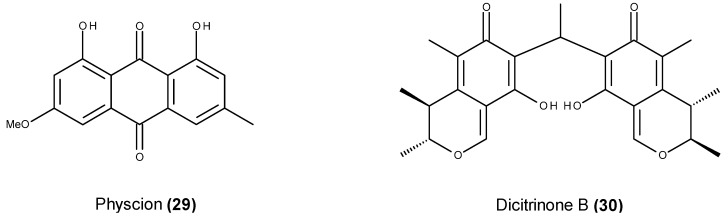
Structure of physcion (**29**) and dicitrinone B (**30**).

In a recent study by Wijesekara *et al.* [90], the anthraquinone physcion (**29**) (Figure 10), which is also known as parietin, was isolated from a culture of the fungus *Microsporum* sp. MFS-YL which was obtained from the surface of the alga *Lomentaria catenata* collected at Guryongpo, PoHang, the Republic of Korea. Physcion (**29**) displayed pro-apoptotic effects in HeLa cells by down-regulating Bcl-2 expression, up-regulating Bax expression, activating the caspase-3 pathway and forming ROS [90].

Dicitrinone B (**30**) (Figure 10), a member of a novel class of carbon-bridged citrinin dimers, was isolated from the marine-derived fungus *Penicillium citrinum* HGY1-5 from crater ash collected from the extinct volcano in Huguangyan, China [91]. This compound inhibited the proliferation of multiple tumor types, including the human A375 melanoma cell line [92]. In addition to inducing G_2_/M phase cycle arrest in the leukemia cell line HL-60 [91], dicitrinone B (**30**) is also a pro-apoptotic agent with effects that are triggered by ROS accumulation and MMPo reduction [92]. Dicitrinone B (**30**) activates both intrinsic and extrinsic apoptosis pathways under the regulation of Bcl-2 family proteins in the human A375 melanoma cell model [92].

**Figure 11:**
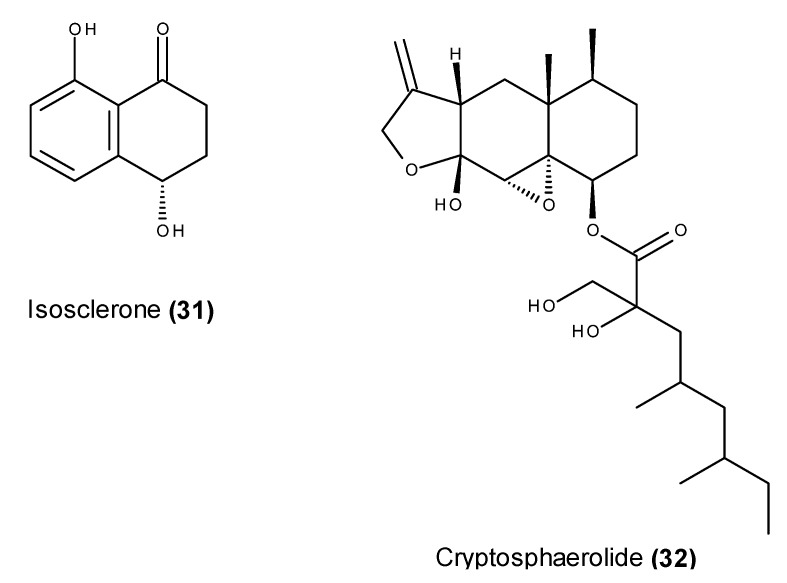
Structure of isosclerone (**31**) and cryptosphaerolide (**32**).

Isosclerone (**31**) (Figure 11) is a naphthalenone that is commonly isolated from marine fungal sources [93,94,95], and it was recently reported in a strain of *Aspergillus fumigatus* obtained from a green alga that was collected at Seosaeng-myeon in the Republic of Korea [96]. Isosclerone-induced pro-apoptotic effects in human breast cancer MCF-7 cells resulted from the down-regulation of Bcl-2 family protein expression, the up-regulation of caspases, and the activation of the NF-κB signaling pathway [97]. This compound also acts on MMP, inhibiting MMP-2 and -9 activities at both the protein and gene levels, and it had a dose-dependent inhibitory effect on the expression of MAPK proteins ERK, JNK and p38 MAPK [96]. Furthermore, this fungal metabolite inhibited cell cycle progression in MCF-7 cells through the down-regulation of CDK2, CDK4, cyclin B1 and cyclin E protein expression [96].

Because of the relevant cytotoxic activity of the crude extract from fungal strain *Cryptosphaeria* sp. CNL-523, which was isolated from an unidentified ascidian collected in the Bahamas, Oh *et al.* [98] performed a chemical investigation leading to the identification of the sesquiterpenoid cryptosphaerolide (**32**) (Figure 11). In addition to its relevant cytotoxic activity against the HCT-116 human colon carcinoma cell line, cryptosphaerolide (**32**) was also identified as an inhibitor of Mcl-1 protein [98], which is a key player in apoptosis [99].

### 2.2. Metabolites That Kill Cancer Cells without Direct Pro-Apoptotic Effects

**Figure 12:**
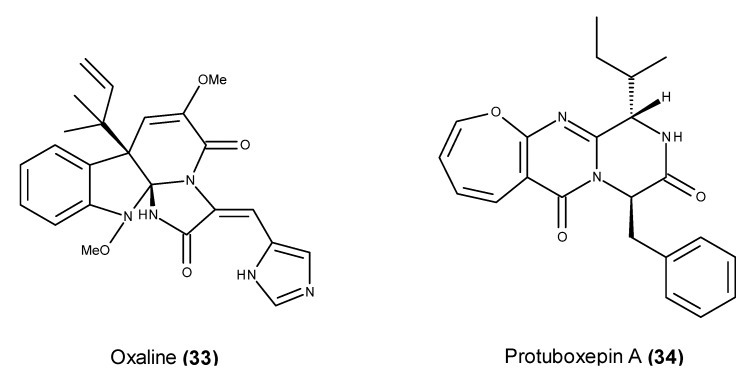
Structure of oxaline (**33**) and protuboxepin A (**34**).

Oxaline (**33**) (Figure 12), the 9-*O*-methyl analog of meleagrin (**16**) (Figure 5), was isolated from the culture of a marine sponge-associated *Penicillium* sp., and also from an unidentified algicolous fungus [100,101]. Analogous to some naturally derived chemotherapeutic agents such as colchicine, vinblastine and taxol, oxaline (**33**) causes cell cycle arrest in the M phase but not G_2_ phase arrest [102]. Oxaline (**33**) inhibits the *in vitro* polymerization of microtubule protein and purified tubulin in a dose-dependent manner. Furthermore, oxaline (**33**) was shown to inhibit the binding of [3H] colchicine to tubulin, but not that of [3H] vinblastine in a binding competition assay [102].

Protuboxepin A (**34**) (Figure 12) belongs to a rare class of naturally occurring diketopiperazine alkaloids containing an oxepin ring system, and it was originally isolated from the EtOAc extract from the culture of *Aspergillus* sp. SF-5044, which was isolated from a sediment sample collected from Dadaepo Beach, Busan, Korea [103]. Recently, protuboxepin A (**34**) was also reported as a product of the co-fermentation of two algicolous fungi of the genus *Aspergillus* (BM-05 and BM-05L) [104]. In addition to its modest growth inhibitory effect against cancer cell lines [103], protuboxepin A (**34**) was further investigated to elucidate its cytotoxic mechanism. The preliminary results revealed increased subG_1_ and G_2_/M phase populations in protuboxepin A-treated Hep3B and MDA-MB-231 cells, in addition to the induction of Bcl-2 (Ser70), the decreased phosphorylation of cdc2 (Tyr15) and the induced cleavage of apoptotic markers in Hep3B cells, suggesting that the cytotoxic activity of protuboxepin A (**34**) was caused by tubulin inhibition [105]. Protuboxepin A’s (**34**) microtubule-stabilizing activity was further confirmed when this metabolite was associated with α/β-tubulin heterodimers, consequently leading to chromosome misalignment in the nuclei via accelerated tubulin polymerization [105]. Furthermore, when using an EB1-EGFP-expressing HeLa cell system, protuboxepin A (**34**) was found to cause a marked and rapid suppression of EB1-EGFP signals, confirming its inhibitory effect on microtubule dynamics [105]. Asami *et al.* [105] described protuboxepin A (**34**) as a microtubule-stabilizing agent, which has a distinct chemical structure from the previously reported microtubule inhibitors, such as taxanes and vinca alkaloids.

**Figure 13:**
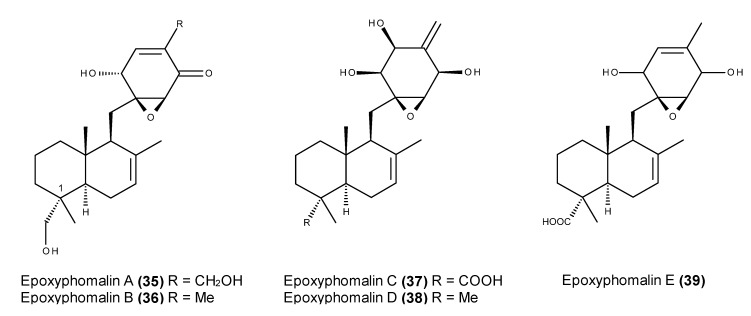
Structures of epoxyphomalins A–E (**35**–**39**).

Epoxyphomalins A–E (**35**–**39**) (Figure 13) belong to a small class of meroterpenoids, which are characterized by a sesquiterpene epoxycyclohexenone skeleton. They were produced by the fungus *Paraconiothyrium cf.* *sporulosum*, which was isolated from the marine sponge *Ectyplasia perox* that was collected at Lauro Club Reef, Dominica [106,107].

Epoxyphomalins A (**35**) and B (**36**) exhibited potent *in vitro* growth inhibitory activity in 12 and 8 of the 36 tested human tumor cell lines [106], respectively, and epoxyphomalin D (**38**) was particularly cytotoxic toward prostate PC3M and bladder BXF1218L cancer cell lines [107]. Apparently, the substituents at C-1 are involved in cytotoxic activity given that epoxyphomalins C (**37**) and E (**39**) (which have a large polar carboxylic acid functionality) displayed significantly lower cytotoxicity [107]. Because of the unique activity profile of the COMPARE analysis, the epoxyphomalin A mode of action (**35**) seemed to be distinct from those of reference anticancer agents [106]. Both epoxyphomalins A (**35**) and B (**36**) displayed potent inhibitions of the proteasome complex, exhibiting the inhibitory activity of chymotrypsin-, caspase-, and trypsin-like proteasomes in the purified human 20S proteasomes incubated with both compounds [107].

**Figure 14:**
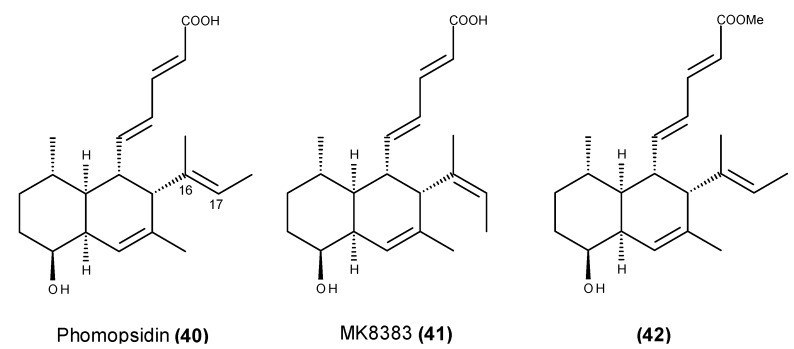
Structure of phomopsidin (**40**), MK8383 (**41**) and phomopsidin methylester derivative (**42**).

Phomopsidin (**40**) (Figure 14) is a potent inhibitor of microtubule assembly, and it was isolated from an extract of *Phomopsis* sp. (strain TUF95F47) that was in turn isolated from a coral reef in Pohnpei [108,109]. Phomopsidin (**40**) inhibits microtubule assembly with an IC_50_ of ~6 μM, which is similar in potency to that of colchicine (IC_50_ = 10 μM) and rhizoxin (IC_50_ = 4 μM) [108,109]. A further antimitotic activity screening of compounds that are structurally similar to phomopsidin (**40**) indicates that the *cis*-decaline structure may play a key role in their antimicrotubule activity [109,110].

The (16*Z*)-isomer of phomopsidin (**40**), which is called MK8383 (**41**) (Figure 14), also displayed potent antimicrotubule activity (IC_50_ = 8 μM), suggesting that the geometry at Δ^16,17^ did not affect the bioactivity, and the lack of activity of the phomopsidin methylester derivative (**42**) (Figure 14) and several structurally related compounds revealed that the free carboxylic acid moiety is essential for the inhibitory activity against microtubule assembly [111,112].

**Figure 15:**
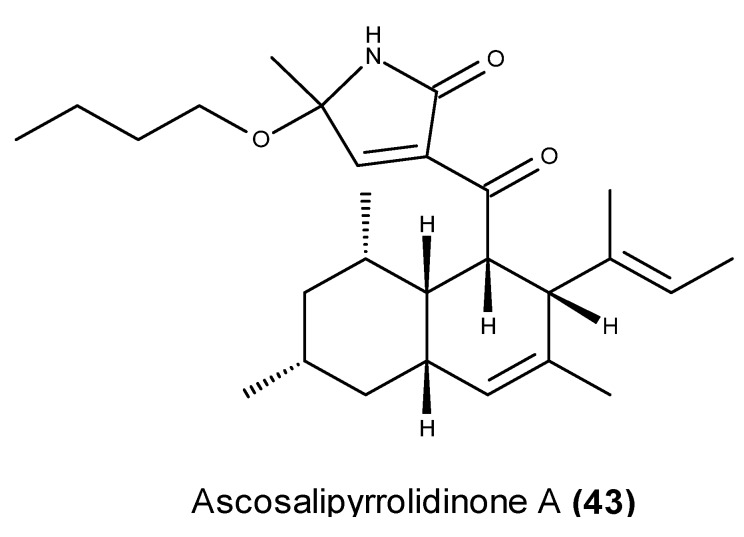
Structure of ascosalipyrrolidinone A (**43**).

Ascosalipyrrolidinone A (**43**) (Figure 15) is another marine fungal-derived tyrosine kinase inhibitor with an unusual tetramic acid skeleton. It was isolated from the EtOAc extract of a culture of the obligate endophytic fungus *Ascochyta salicorniae* that was obtained from a green alga *Ulva* sp. collected off the North Sea by Tӧnning in Germany [113]. This compound caused 70% activity inhibition of p56*^lck^* at the concentration of 40 μg/mL [113].

**Figure 16:**
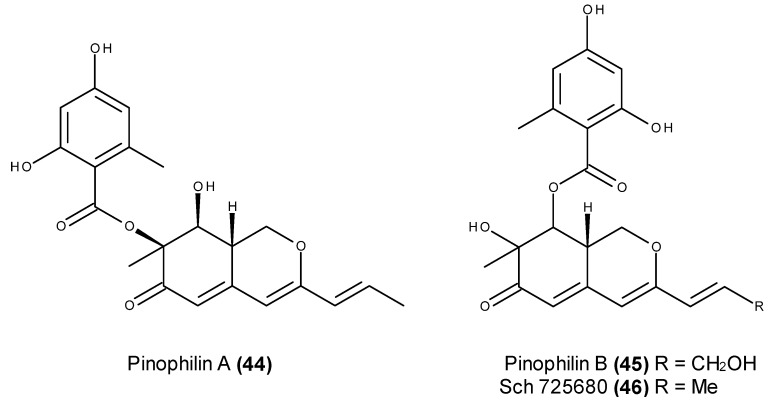
Structure of pinophilin A (**44**), pinophilin B (**45**) and Sch 725680 (**46**).

Three hydrogenated azaphilones called pinophilins A (**44**) and B (**45**) and Sch 725680 (**46**) (Figure 16) were isolated from cultures of the fungus *Penicillium pinophilum* Hedgcok. This fungus was derived from seaweed collected along the coast of Kasai Marine Park in Tokyo, Japan [114]. These fungal metabolites displayed inhibitory activities against mammalian DNA polymerases (pols) A (pol *γ*), B (pols *α*, *δ*, and *ε*), and Y (pols *η*, *ι*, and *κ*) families, but they had no inhibitory activity against the four X-family pols (pols *β*, *λ*, *μ*, and terminal deoxynucleotidyl transferase) [114]. Additionally, no activity or only a minimal influence was detected on the activities of plant and prokaryotic pols or any other DNA metabolic enzymes, indicating specific inhibitory activities for A-, B-, and Y-family pols. Pinophilin A (**44**) displayed the strongest inhibitory activity on the DNA pols, and it exhibited a noncompetitive inhibitory activity against both pols *α* and *κ* with the DNA template-primer substrate, and competitive inhibitory activity with the nucleoside substrate [114]. Furthermore, pinophilins A (**44**) and B (**45**) and Sch 725680 (**46**) suppressed cell proliferation and growth in five human cancer cell lines, leading to cell cycle arrest at the S phase in BALL-1 and HCT116 cells, with no cytotoxicity against normal human cell lines [114]. The correlation between the inhibitory effects of cancer cell growth and the inhibition of B-family pols, which are essential for DNA replication during cell proliferation, indicate that the anticancer activity of these compounds was caused by the inhibition of DNA replication [114].

**Figure 17:**
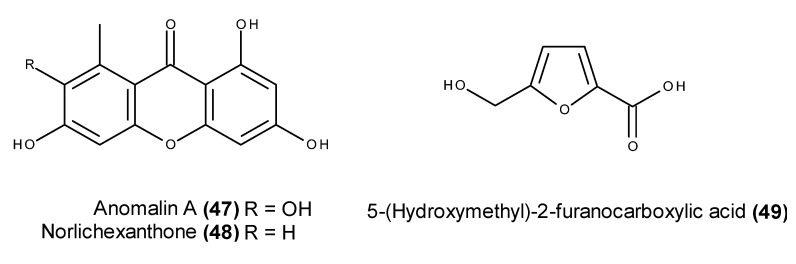
Structure of anomalin A (**47**), norlichexanthone (**48**) and 5-(hydroxymethyl)-2-furanocarboxylic acid (**49**).

A chemical investigation of the EtOAc extract of a culture of the algicolous fungus *Wardomyces anomalus*, which was collected around Fehmarn Island in the Baltic Sea, resulted in the isolation of several xanthones, including anomalin A (**47**) and norlichexanthone (**48**), as well as 5-(hydroxymethyl)-2-furanocarboxylic acid (**49**) (Figure 17) [115]. All these compounds caused 100% inhibition of p56*^lck^* tyrosine kinase at 200 μg/mL [115].

Anomalin A (**47**) and norlichexanthone (**48**) were later reported in the cytotoxic extract of the fungus *Arthrinium* sp., which was isolated from the marine sponge *Geodia cydonium* that was collected from the Adriatic Sea [116]. In addition to their strong cytotoxic activity against a panel of human and murine cell lines, anomalin A (**47**) and norlichexanthone (**48**) also exhibited inhibitory activity against several cancer-related protein kinases. Anomalin A (**47**) and norlichexanthone (**48**) markedly inhibited the activity of the protein kinases aurora-B, PIM1 and VEGF-R2 with mean IC_50_ values ranging from 0.3 to 0.8 μM and 0.3 to 12 μM, respectively [116].

**Figure 18:**
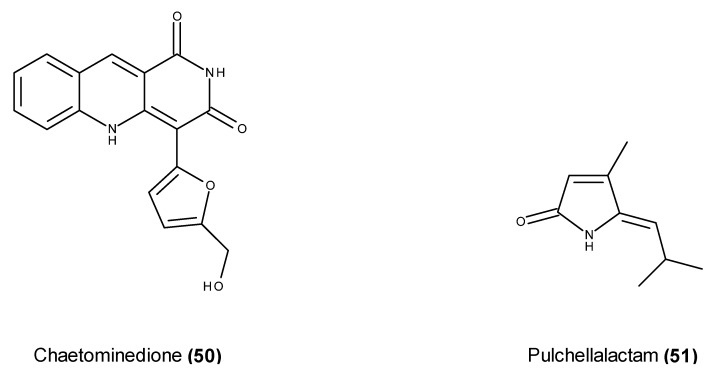
Structure of chaetominedione (**50**) and pulchellalactam (**51**).

Chaetominedione (**50**) (Figure 18) is a benzonaphthyridinedione derivative, and it was isolated from an extract of the fungus Chaetomium sp., which was obtained from *Valonia utricularis* alga that was collected from Azores, Portugal [117]. This compound has significant inhibitory activity toward p56^lck^ tyrosine kinase (94% enzyme inhibition at 200 μg/mL) [117], which is the enzyme that is involved in tumor cell biology under hypoxic environments [118].

Pulchellalactam (**51**) (Figure 18) was isolated from the EtOAc extract of a culture of the driftwood marine fungus *Corollospora pulchella* ATCC 62554, which was collected from Peleliu, Palau [119]. Pulchellalactam (**51**) exhibited dose-dependent inhibitory activity against the CD45 protein tyrosine phosphatase, which plays central signaling roles in dephosphorylating Src-kinases, and this compound therefore represents an attractive drug target [119].

**Figure 19:**
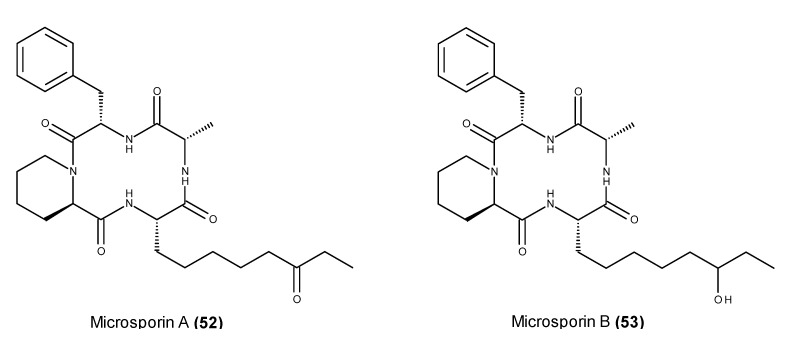
Structure of microsporin A (**52**) and B (**53**).

A bioassay-guided fractionation of the EtOAc extract from a culture of the fungus *Microsporum cf. gypseum* isolated from a bryozoan *Bugula* sp. that was collected in the Virgin Islands yielded two cyclic tetrapeptides known as microsporins A (**52**) and B (**53**) (Figure 19) [120]. Both peptides displayed strong cytotoxic activity against human colon adenocarcinoma HCT-116 cells; however, microsporin B (**53**) showed reduced activity (IC_50_ = ~9 μg/mL) compared with microsporin A (**52**) (IC_50_ value of 0.6 μg/mL), suggesting that the carbonyl group of the oxodecanoic acid moiety may influence the cytotoxicity [120].

Microsporin A (**52**) also exhibited significant cytotoxicity against an NCI 60 cancer cell panel with a mean IC_50_ of ~3 μM [120]. This compound displayed potent *in vitro* inhibitory activity against a mixture of HDACs and HDAC8, exhibiting stronger activity than the anticancer HDAC agent vorinostat [120].

**Figure 20:**
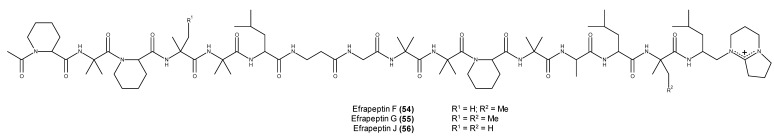
Structure of efrapeptin F (**54**), G (**55**) and J (**56**).

A chemical investigation of the fungus *Tolypocladium* sp. AMB18, which was isolated from sea mud collected in Aomori prefecture, Japan, resulted in the isolation of thee linear pentadecapeptides called efrapeptins F (**54**), G (**55**) and J (**56**) [121] (Figure 20).

These peptides inhibited the luciferase expression caused by 2-deoxyglucose in HT1080 human fibrosarcoma cells, with IC_50_ values of ~9, ~3 and 18 nM, respectively. Efrapeptin J (**56**) also acts as a down-regulator of the molecular chaperone GRP78 in HT1080 and MKN74 human gastric cancer cells, and it induced cell death in HT1080 cells under endoplasmic reticulum stress [121]. Molecular chaperone GRP78 is an important target for the development of anticancer agents because of its key roles in cancer cell biology, namely in chemoresistance [122,123].

**Figure 21:**
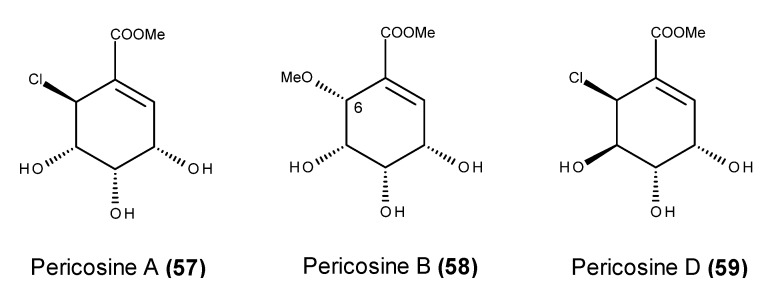
Structure of pericosine A (**57**), B (**58**) and D (**59**).

### 2.3. Metabolites with in Vivo Antitumor Activity or Those Entering Clinical Trials

Pericosines A (**57**), B (**58**) and D (**59**) (Figure 21) are cytotoxic metabolites that belong to a small class of cyclohexenoids produced by the fungal strain *Periconia byssoides* OUPS-N133 which was isolated from the gastrointestinal tract of the sea hare *Aplysia kurodai* [124,125]. The three fungal metabolites exhibited pronounced *in vitro* cytotoxic activity against murine P388 cells, and pericosine A (**57**) also displayed selective and potent cytotoxicity against HBC-5 and SNB-75 human cancer cell lines [125]. Interestingly, when compared with natural pericosine B (**58**), its synthetic C-6 epimer displayed significantly weaker cytotoxicity against the murine P388 cell line, suggesting that the stereochemistry of C-6 may have a preponderant role in pericosine B (**58**) activity [126].

In addition to its inhibitory activity against protein kinase EGFR and human topoisomerase II [125], pericosine A (**57**) displayed significant *in vivo* inhibitory activity in mice that were inoculated intraperitoneally (i.p.) with P388 leukemia cells, leading to increased survival days in mice administered i.p. with 25 mg/kg of the metabolite [124,125]. It should nevertheless be emphasized that although murine cancer models are useful for studying the *in vivo* anticancer effects of various types of compounds, they are actually not representative of the biology of human cancers in a clinical setting [127].

**Figure 22:**
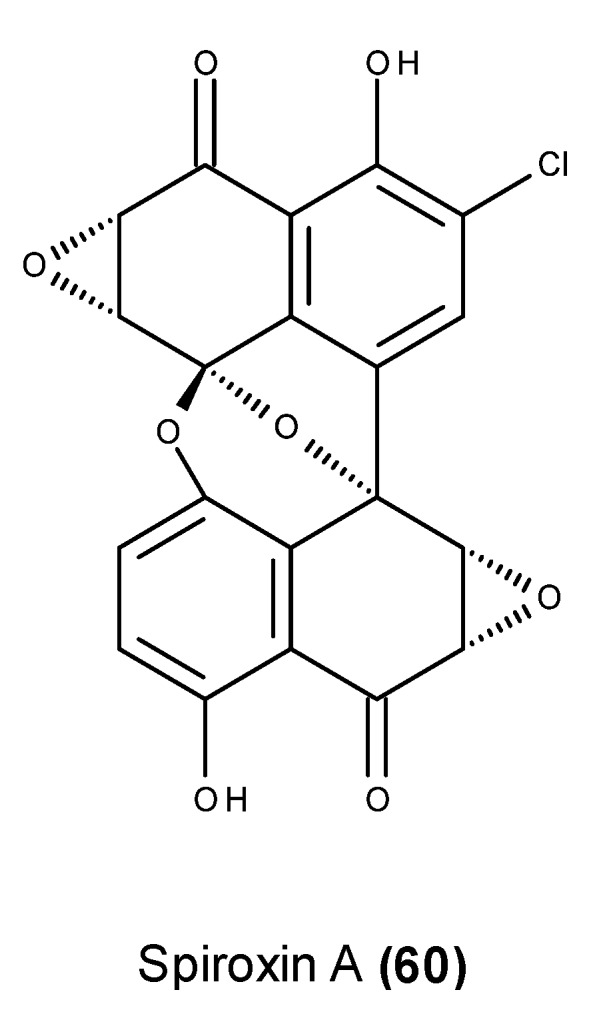
Structure of spiroxin A (**60**).

Spiroxin A (**60**) (Figure 22), which is a cytotoxic antibiotic, was produced by an unidentified fungus (strain LH-37H248) isolated from a soft coral that was collected from Dixon Bay, Canada [128]. Spiroxin A (**60**) displayed potent cytoxicity (IC_50_ = 0.09 μg/mL) against a panel of 25 diverse cell lines, which were supposedly partially derived from single-stranded DNA cleavage [128]. Spiroxin A (**60**) contains an unusual bisnaphthospiroketal octacyclic ring system with the spiroketal carbon oxidation state, allowing it to behave chemically as a quinone epoxide and leading to DNA cleavage via an oxidative stress mechanism involving thiol conjugates [128].

Spiroxin A (**60**) also displayed *in vivo* antitumor activity in nude mice against ovarian carcinoma, causing 59% inhibition after 21 days at a 1 mg/kg dose administered i.p. on days 1, 5, and 9 post-staging [128]. The fact that ovarian cancers do not develop subcutaneously in clinical situations [129] makes it difficult to assess the actual value of this *in vivo* assay because there was no indication as to whether the ovarian cancer was grafted subcutaneously (s.c.) or orthotopically.

**Figure 23:**
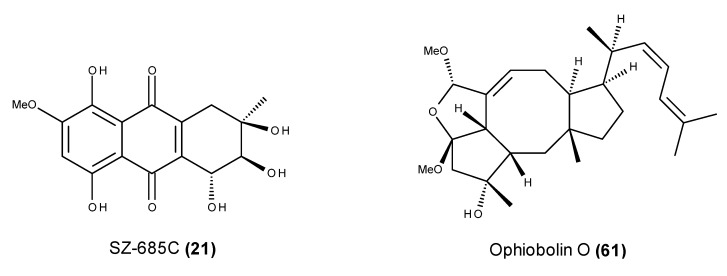
Structure of SZ-685C (**21**) and ophiobolin O (**61**).

Breast cancer remains one of the most common cancers, and anthracyclines, including adriamycin, are commonly used as breast cancer chemotherapeutic drugs [130,131,132]. The HER2/PI3K/Akt signaling pathway is one of the multidrug-related pathways, and it is involved in breast cancer chemoresistance against a panel of anticancer drugs such as the anthracyclines adriamycin, epirubicin and mitoxantrone [133]. The previously mentioned pro-apoptotic SZ-685C (**21**) (Figure 23), which displayed inhibitory activity against the proliferation of six human breast cancer cell lines [78], also markedly inhibited tumor growth in nude mice that were inoculated s.c. in the right mammary gland with human breast MDA-MB-435 cancer cells. With an i.p. dose of 50 mg/kg body weight, this compound led to 61% inhibition in the xenografted tumor growth after 35 days, with no detectable toxicity [78]. SZ-685C (**21**) also caused a significant inhibition in the growth of MCF-7/ADR xenografted tumors in nude mice. In contrast to the reference breast chemotherapeutic agent adriamycin, which causes a certain degree of toxicity as well as significantly lower inhibitory activity (28%) [80], the i.p. administration of SZ-685C (**21**) at 50 mg/kg body weight caused 64% inhibition in tumor growth with no detectable toxic effects.

Ophiobolin O (**61**) (Figure 23), which is a sesterterpene with an unusual tetracyclic ring system that was isolated from *Aspergillus ustus* 094102, was reported as pro-apoptotic in human MCF-7 breast cancer cells [134]. Ophiobolin O (**61**) causes the activation of JNK, p38 MAPK and ERK, as well as the degradation of Bcl-2 phosphorylation (Ser70) [134]. Ophiobolin O (**61**) also decreases the phosphorylation level of AKT and GSK3β, as well as the induction of cyclin D1 down-regulation [135]. Furthermore, this compound was found to reverse MCF-7/ADR resistance to adriamycin [136], and its reversal effect may be related to the reduction in resistance-related protein P-glycoprotein (P-gp, also known as MDR1) by inhibiting the activity of the multidrug resistance 1 (MDR1) gene promoter, which makes MCF-7/ADR cells more sensitive to adriamycin treatment [136]. Ophiobolin O was also found to suppress tumorigenesis in the xenograft mouse model that was injected s.c. with MCF-7 cells. The intravenous (i.v.) administration of 5, 10 and 20 mg/kg of ophiobolin O (**61**) led to 35%, 46% and 69% tumor growth inhibitory rates, respectively, after 50 days of treatment, displaying almost no toxicity [136]. At a dose of 20 mg/kg, the compound showed nearly equivalent inhibitory activity to the activity in a paclitaxel-treated mouse, which caused 73% tumor growth inhibition [135]. Although the i.v. administration of 5 mg/kg ophiobolin O (**61**) and adriamycin alone produced MFC-7/ADR tumor growth inhibitory rates of 23% and 46% in a nude mice xenograft model, respectively, the combined treatment (5 mg/kg) revealed a strong reversal effect leading to an inhibition rate of 71% [136]. The improved antitumor effect of adriamycin, in combination with ophiobolin O (**61**), confirmed its potential as a lead candidate to reverse chemotherapy drug resistance in breast cancer [136].

**Figure 24:**
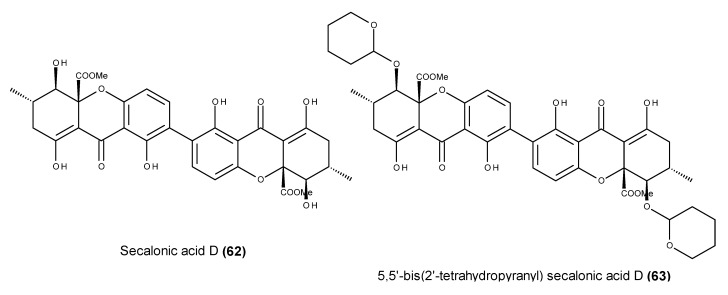
Structure of secalonic acid D (**62**) and its synthetic derivative (**63**).

Secalonic acid D (**62**) (Figure 24) is a toxic and teratogenic dimeric xanthone that has been widely reported in marine-derived fungal strains [137,138,139,140,141]. It exhibited an inhibitory effect on the K562 leukemia cell line, causing a concentration-dependent block in the G_0_/G_1_ cell cycle phase [137,138,142] as well as apoptosis induction and cell cycle arrest in the G_1_ phase in K562 and HL60 cells [143]. Although the apoptotic effect apparently derives from the cleavage of caspase-3 and PARP, the cell cycle arrest in the G_1_ phase seems to be related to the down-regulation of the proto-oncogene c-Myc [138]. The decreased expression of c-Myc and the subsequent down-regulation of cyclin D1, CDK4 and E2F1 by secalonic acid D (**62**) apparently derive from the activation of p-GSK-3α/β following the breakdown of β-catenin [138]. In addition to its potent cytotoxic effect on MDR and parental cells, secalonic acid D (**62**) also exhibited remarkable cytotoxicity against lung cancer cells A549, GLC82 and H460, with IC_50_ values of 0.3, 0.3 and 0.1 μM, respectively [143]. Secalonic acid D (**62**) was also evaluated for its activity against the overexpression of ABCG2 protein, which is the major mechanism responsible for chemotherapy resistance in S1-M1-80 cells [144], and it was found to quicken the degradation of ABCG2 through the activation of calpain 1 in S1-M1-80 cells. This effect is supposedly related to the decreased percentage of side population cells, which are resistant to chemotherapeutic agents such as doxorubicin and cisplatin [143]. Secalonic acid D (**62**) was also identified as a topoisomerase I inhibitor. Unlike camptothecin, the lead metabolite of the anticancer drugs irinotecan and topotecan, secalonic acid D (**62**) was found to hamper the formation of topoisomerase I-DNA covalent complexes [145]. A further evaluation of the secalonic acid D (**62**) topoisomerase I inhibition mechanism by docking studies revealed its strong binding activity with the catalytic residues ARG488 and LYS532, as well as with ARG590 and TYR723 through its two carbonyl groups and hydroxyl moieties [141]. These results indicated that secalonic acid D (**62**) inhibited DNA molecules from binding with the catalytic residues or even the catalytic region [141]. The *in vivo* antitumor activity of secalonic acid D (**62**) in mouse hepatoma H22-inoculated mice xenografts was also evaluated [141]. This compound was found to inhibit tumor cell growth at an inhibitory rate that was significantly higher than that of 5-fluorouracil (5-FU) (42% *versus* 38%) at 15 and 20 mg/kg, respectively. Furthermore, when compared with 5-FU, the weight of secalonic acid D-treated mice did not change significantly, indicating the lower toxicity of secalonic acid D (**62**) [141]. The *in vitro* and *in vivo* antitumor effect of the 5,5′-bis(2′-tetrahydropyranyl) derivative of secalonic acid D (**63**) was also assayed [146]. The synthetic derivative (**63**) (Figure 24) exhibited *in vitro* inhibitory activity similar to that of mitomycin C against syngeneic fibrosarcoma Meth-A cells. This compound also showed *in vivo* antitumor activity against BALB/c mice bearing Meth-A cells, supposedly through direct interactions with the Meth-A cells [146].

**Figure 25:**
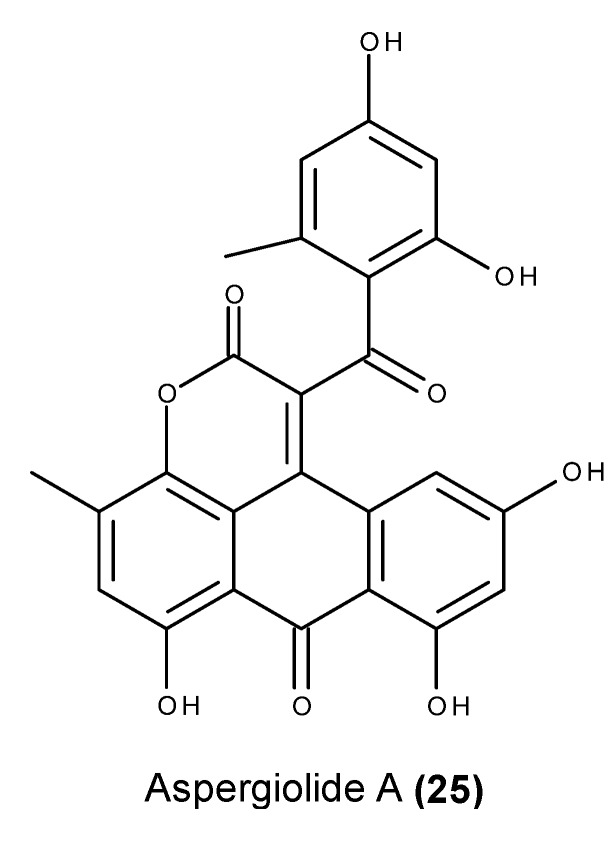
Structure of aspergiolide A (**25**).

Aspergiolide A (**25**) (Figure 25) also displayed significant *in vivo* antitumor activity in both H22 and BEL-7402 hepatocellular carcinoma xenografts [89]. The i.p. administration of aspergiolide (**25**) significantly inhibited the growth of H22 subcutaneous cancer with a higher inhibitory rate and lower toxicity than that of adriamycin. I.p. administration of aspergiolide A (**25**) resulted in relevant anticancer activity in BEL-7402 xenografts; however, the inhibitory rate and impact of aspergiolide A (**25**) on mouse body weight reduction were lower than the reductions in ADR-treated mice [89].

An early safety evaluation of aspergiolide A (**25**) revealed maximum tolerable doses in mice that were superior to 400 mg/kg, exceeding the effective dose 10 times. Because no significant increase in micronucleus rates or inhibitions of the hERG channel was observed, the compound was not considered to be genotoxic or cardiotoxic [89]. Furthermore, an evaluation of pharmacokinetic parameters and tissue distribution indicated that aspergiolide A (**25**) could be rapidly cleared *in vivo* in a first-order reaction kinetic manner, and the compound was enriched in cancer tissue [89]. Moreover, the compound was taken up well by Caco-2 cells and absorbed through the active transport pathway [89].

**Figure 26:**
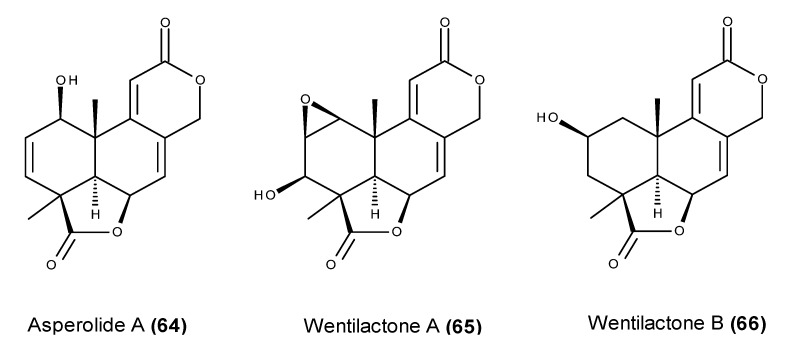
Structure of asperolide A (**64**), wentilactone A (**65**) and B (**66**).

An extract of the endophytic fungus *Aspergillus wentii* EN-48 that was isolated from marine alga *Sargassum* sp. displayed relevant cytotoxic activity against several human tumor cell lines, leading to the chemical investigation of its bioactive metabolites [147]. The fungus was found to produce asperolide A (**64**) (Figure 26) in addition to the structurally related norditerpene dilactones called wentilactones A (**65**) and B (**66**) (Figure 26) [147]. Despite their modest cytotoxic activity against a panel of human tumor cell lines, these compounds inhibited the proliferation of human cancer cells by G_2_/M arrest as well as by inducing apoptosis [148,149]. Asperolide A (**64**) exerted an antiproliferative effect on human NCI-H460 lung carcinoma cells through the activation of the Ras/Raf/MEK/ERK signaling and p53-dependent p21 pathways [148]. Similarly, wentilactone A (**65**) directly targeted HRas-GTP, excessively activating the Ras/Raf/ERK pathway and inducing apoptosis and cell cycle arrest in both NCI-H460 and NCI-H446 lung cancer cell lines [149]. The apoptotic effect of wentilactone A (**65**) is also apparently mediated by ROS accumulation [149]. Wentilactone B (**66**) also markedly induced cell cycle arrest at the G_2_ phase and mitochondrial-related apoptosis, accompanying the accumulation of ROS [150]. A pre-treatment of human hepatoma SMMC-7721 cells with wentilactone B (**66**) revealed a specific binding affinity toward Ras-GTP, suggesting that the compound causes cell cycle arrest in the G_2_ phase through the Ras/Raf/ERK signaling pathway, and it induces apoptosis via the Ras/Raf/JNK pathway [150]. Furthermore, wentilactone B (**66**) not only displayed a selective cytotoxic effect on SMMC-7721 cells, but also inhibited the metastasis of SMMC-7721 cells through the down-regulation of cell surface proteins CD44 and EGFR [151].

In addition to their cytotoxic activity against a wide panel of cancer cell lines, asperolide A (**64**), wentilactone A (**65**) and wentilactone B (**66**) were able to suppress the *in vivo* tumor growth of in-mouse xenografted models [148,149,150]. The treatment of nude mice inoculated with NCI-H460 lung cancer cells with asperolide A (**64**) i.v. at 2.5 mg/kg promoted both tumor mass and volume reduction, causing a 68% inhibition of tumor growth. Unlike cisplatin-treated mice, which showed an inhibition ratio that was 14 times higher, asperolide A (**64**) led to weight gain, showing little toxicity compared with cisplatin [148]. Similar to asperolide A (**64**), wentilactone A (**65**) also displayed relevant *in vivo* inhibitory activity against NCI-H460 and NCI-H466-xenografted mice. *In vivo* wentilactone A (**65**) treatment caused an effective inhibitory effect in both xenografts with inhibition rates of 96% and 87%, respectively, showing effects equivalent to that of cisplatin i.v. at 2.5 mg/kg, but promoting weight gain over the course of the therapy [149]. Consistent with the *in vitro* anticancer mechanism, a tumor immunohistochemical analysis revealed a relevant increase in active caspase-3 and the inhibition of cdc2 expression in wentilactone A-treated mice, in addition to increased levels of p-ERK and HRas-GTP, indicating the same cell signaling mechanism [149]. An *in vivo* antitumor activity evaluation revealed that the i.p. administration of wentilactone B (**66**) inhibited the growth of transplanted human hepatoma SMMC-7721 cells in a dose-dependent manner, leading to an 86% inhibition of tumor growth at a dose of 20 mg/kg [150]. Despite its significantly higher *in vivo* antitumor activity, 5-FU treatment caused a large body weight decrease in contrast to wentilactone B-treated animals, thus evidencing the lower toxicity of wentilactone B (**66**) [150]. A further analysis of wentilactone B-treated xenograft tissues revealed the up-regulation of Ras-GTP as well as the activation of ERK and JNK, confirming the involvement of the MAPK pathway in the wentilactone B (**66**) anticancer effect [150].

**Figure 27:**
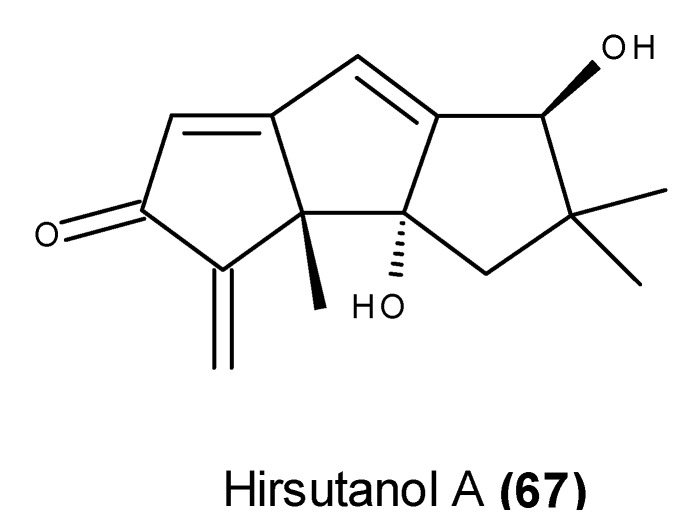
Structure of hirsutanol A (**67**).

Hirsutanol A (**67**) (Figure 27), a hirsutane-based sesquiterpene, was first reported in the salt water culture of an unidentified marine sponge-associated fungus (95-1005C) [152]. Hirsutanol A (**67**), as well as other derivatives of this class of sesquiterpenes, was later isolated from the EtOAc extract of a culture of the fungus *Chondrostereum* sp. SF002, which was isolated from the soft coral *Sarcophyton tortuosum* that was collected from Sanya Bay in the southern China Sea [153,154]. Hirsutanol A (**67**) was found to inhibit cell proliferation, elevate the ROS level, and induce apoptosis and autophagy in MCF-7 human breast cancer cells [155]. It therefore seems that hirsutanol A (**67**) could induce apoptosis and autophagy via the accumulation of ROS, and co-treatment with an autophagy inhibitor was able to sensitize MCF-7 cells to hirsutanol A (**67**) [155]. Hirsutanol A (**67**) also activates the JNK signaling pathway by elevating the ROS level [156]. In contrast with other hirsutane sesquiterpenoids, which lack the *α*-methylidene oxo group, hirsutanol A (**67**) displayed a potent cytotoxicity against 15 distinct human cancer cell lines [153]. These results indicated that the α-methylidene oxo group is required for the cytotoxic activity of this class of sesquiterpenoids, possibly by enzymatic inactivation via alkylation [153].

Hirsutanol A (**67**) also exhibited an *in vivo* anti-tumor effect in a SW620 xenograft model, causing more than 50% inhibition in tumor growth at 10 mg/kg/day when administered i.p. [156].

**Figure 28:**
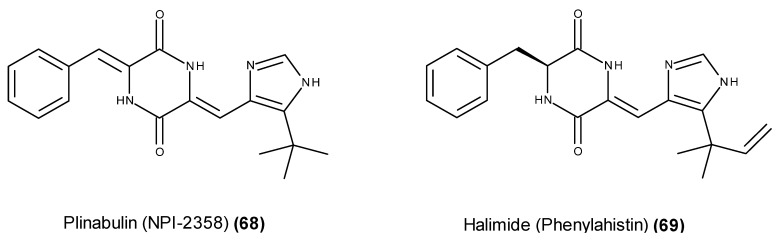
Structure of plinabulin (**68**) and halimide (**69**).

Plinabulin (NPI-2358) (**68**) (Figure 28), a selective tumor vascular disrupting agent, is the only marine-derived fungal metabolite-based compound that has entered clinical trials to date. Plinabulin (NPI-2358) (**68**) is a synthetic *tert*-butyl analog that is closely related to the naturally occurring fungal metabolite halimide (**69**) (Figure 28), also known as phenylahistin [157,158]. Halimide (**69**) was isolated from cultures of the strain *Aspergillus* sp. CNC139, which was collected off the Philippine Islands [157,158], and almost simultaneously from two terrestrial strains of *Aspergillus ustus* [159].

In a panel of 38 human tumor cell lines, halimide (**69**) exhibited an *in vitro* growth inhibition profile that was similar to that of the vinca alkaloids or paclitaxel, suggesting that its target of action could be the microtubule system [159]. This finding was later confirmed by its inhibitory activity on the cell cycle progression of P388 cells in the G_2_/M phase [160] and the disruption of the microtubule network in A549 cells through interactions with the colchicine-binding site on tubulin [161]. Its promising *in vivo* antitumor activity against P388 leukemia cells and solid tumors of Lewis lung carcinoma [162] motivated the synthesis of several halimide analogs, and a cytotoxic activity evaluation led to the development of plinabulin (NPI-2358) (**68**) [163,164]. Plinabulin (**68**) displayed a potent *in vitro* antitumor activity against several human tumor cell lines including MDR cell lines [165], and similar to halimide (**69**), plinabulin was also identified as a potent microtubule-disrupting agent with colchicine-like tubulin-depolymerizing activity [166]. Additionally, plinabulin (**68**) induced monolayer permeability in normal human umbilical vein endothelial cells (HUVECs), displaying significantly more potent vascular disrupting activity than the tubulin-depolymerizing agents colchicine and vincristine [165]. Plinabulin (**68**) displays microtubule-disrupting and vascular disrupting activities, in addition to the tumor growth inhibitory activity of other preclinical models, and together with an ADMET parameter evaluation, it was classified as a clinical candidate [167]. A phase I first-in-human trial sponsored by Nereus Pharmaceuticals Inc. was performed to examine its safety, pharmacokinetics and pharmacodynamics as a single agent in patients with refractory solid tumors or lymphoma [168,169]. Phase I/II clinical trials of plinabulin (**68**) in combination with docetaxel was completed in 2011 in patients with advanced non-small cell lung cancer [170,171,172].

## 3. Which Compounds Are the Most Promising Marine-Derived Fungal Metabolites for Use as Potential Anticancer Agents?

Gliotoxin (**7**), 5a,6-didehydrogliotoxin (**8**) and gliotoxin G (**9**) exhibit potent inhibitory activity against the methylation regulation enzymes HMT G9a and HMT Set7/9 [53], which are commonly involved in the malignant transformation of cells [58]. Kondo *et al.* [173] report that the knockdown of HMT G9a in human PC3 prostate cancer cells markedly inhibited cell growth and caused profound morphological changes with a loss of telomerase activity and shortened telomeres. Kondo *et al.* [173] also reported that HMT G9a is required to perpetuate the malignant phenotype. These authors [173] accordingly claim that targeting HMT G9a may be of therapeutic benefit for cancers. Lee *et al.* [174] showed that hypoxia results in the up-regulation of HMT G9a, and the overexpression of HMT G9a attenuates RUNX3 expression, along with the fact that RUNX3 is a tumor suppressor [174]. These authors [174] thus argue that hypoxia silences RUNX3 by epigenetic histone regulation during the progression of gastric cancer. HMT G9a is also overexpressed in pancreatic adenocarcinoma, in which it promotes tumor invasiveness and metastasis [175]. BRD4770, which is a small-molecule inhibitor of HMT G9a, induces senescence in PANC-1 pancreatic cancer cells [175]. Adachi *et al.* [176] suggest that T-cadherin might inhibit tumor progression through multiple pathways including the HMT Set7/9-p53 pathway. It therefore seems to us that gliotoxin and some of its metabolites are actually potential anticancer drugs due to their anti-HMT G9a and Set7/9 activities, which represents an innovative mechanism of anti-cancer action.

Bostrycin (**20**) and its analogs represent an interesting family of anticancer compounds for several reasons. Bostrycin induces apoptosis in A549 non-small cell lung cancer (NSCLC), and A549 cancer cells display certain levels of resistance to pro-apoptotic stimuli [177]. In the same manner, SZ-685C (**21**), a tautomer of bostrycin (**20**), is active against radioresistant nasopharyngeal cancer cells [79] and adriamycin-resistant breast cancer cells [80], and 1403P-3 (**22**), a bostrycin derivative (**22**), is also cytotoxic against MDR cancer cells [82]. In addition, although bostrycin (**20**) and its derivatives do not seem to be selective *in vitro* [77], SZ-685C (**21**) displays significant *in vivo* antitumor activity against adriamycin-resistant human breast xenografts with no detectable toxicity at effective doses in terms of anticancer activity [78,80]. All these features thus strongly suggest that bostrycin and its derivatives could be used as scaffolds to generate innovative anticancer agents.

Pinophilin A (**44**) is a selective compound, *i.e.*, it is cytotoxic against cancer but not against normal cells [114]. In addition to its selectivity between normal and cancer cells, pinophilin A (**44**) also displays a rather original mechanism of action through its inhibitory effects against specific mammalian DNA polymerases. This compound could therefore lead to innovative anticancer agents.

Ophiobolin O (**61**) displays several mechanisms of action when killing cancer cells, including for example the inhibition of the activity of several kinases [134,135,136]. It is interesting to note that ophiobolin O (**61**) induces apoptosis in human MCF-7 breast cancer cells [134], and MCF-7 breast cancer cells are deficient in caspase-3 (a key player in apoptosis) activity [178]. A close analog of ophiobolin O, *i.e.*, ophiobolin A, kills glioblastoma cells that are apoptosis-resistant [179] through paraptosis-related events [180]. Ophiobolin O (**61**) also reverses the resistance of human breast cancer cells to adriamycin [136], and it displays *in vivo* antitumor activity in human MCF-7 breast xenografts [136]. All these data strongly suggest that some ophiobolin derivatives could be used to combat various types of cancers that display various levels of resistance to pro-apoptotic stimuli.

## 4. How Could We Increase the Rate of Discovery of Marine-Derived Fungal Metabolites as Potential Anticancer Agents, at Least *in Vitro*?

### 4.1. Are Pro-Apoptotic Compounds Still Valuable Weapons for Combating Cancer?

Several cytotoxic marine-derived fungal metabolites that exhibit pro-apoptotic effects against cancer cells induce apoptosis through the inhibition of the Akt pathway as evidenced, for example, by leptosin C (**3**) [49], SZ-685C (**21**) [78] and ophiobolin O (**61**) [135]. The problem is that hundreds of articles have already described the use of Akt inhibitors to combat various types of cancers, and several Akt inhibitors are already in clinical trials [181,182,183,184]. It thus seems unlikely that compounds for which some *in vitro* data exist today with respect to their Akt inhibition activity would move toward clinical trials in the coming years with respect to this more-than-competitive area of Akt inhibitors.

Another problem relating to the determination of the potential anticancer activity of marine-derived fungal metabolites is as follows. Many studies that emphasize the potential activity of marine-derived fungal metabolites are often obtained by using only one or two cancer cell lines, which are unfortunately sensitive-to-highly sensitive to pro-apoptotic stimuli. This is the case, for example, for fumigaclavine (**19**) [70], isosclerone (**31**) [96], ophiobolin O (**61**) [134] and hirsutanol A (**67**) [155], the anticancer activities of which were primarily characterized in the human MCF-7 breast cancer cell line, which is more or less sensitive to pro-apoptotic stimuli [178]. As by March 2015, the Scopus database includes more than 6400 publications for the keywords “*MCF-7* AND *apoptosis.*” The HL-60 leukemia cell line is also used to characterize the mechanisms of action of marine-derived fungal metabolites for shearinine A (**10**), (**11**) and (**12**) [60,61], as well as for meleagrin D (**14**) and E (**15**) [62]. The Scopus database includes more than 5300 publications for the keywords “*HL-60* AND *apoptosis,*” as of March 2015. Finally, the HeLa cervix carcinoma cell line is also used to characterize the potential anticancer activity of marine-derived fungal metabolites for gliotoxin (**7**) [55], neoechinulin A (**18**) [69], and physcion (**29**) [90]. The Scopus database includes more than 9700 publications for the keywords “*HeLa* AND *apoptosis,*” as of March 2015. Thus, the data that were obtained for cancer cell lines that are sensitive to highly sensitive to pro-apoptotic stimuli are at odds with respect to the clinical situation. In fact, cancers from patients with dismal prognoses are actually resistant to pro-apoptotic stimuli [179,185,186,187,188,189,190,191]. Thus, MCF-7, HeLa and/or HL60 cells cannot be used as predictive models with respect to cancer patients, and the fact that a given compound induces apoptosis in these MCF-7, HeLa and/or HL60 cells definitively does not mean that it is a promising anticancer agent.

Pro-apoptotic agents that can be classified as promising anticancer agents must display pro-apoptotic effects against cancer cells that usually resist pro-apoptotic insults. Compounds that kill MDR and apoptosis-resistant cancer cells through non-apoptotic pathways should be privileged [42].

### 4.2. Pharmacological and Toxicological Strategies

When assayed *in vitro*, a compound that kills cancer cells is not a potential anticancer agent; it is just a poisonous compound. To become a potential anticancer agent, a cytotoxic compound must kill more cancer cells than normal cells. Thus, once a compound displays *in vitro* cytotoxic activity against cancer cells, it must be tested as soon as possible on normal cells to determine its level of selectivity between normal and cancer cells. Thus, if a compound of interest displays the same levels of toxicity in normal and cancer cells, it cannot be labeled as a potential anticancer agent. It is just a non-selective poisonous agent [42].

If a compound that is assayed *in vitro* displays cytotoxicity against various cancer cell lines while still being selective towards normal cells, e.g., it is significantly less toxic against normal than cancer cells, this selective cytotoxic compound must then be assayed against several MDR cancer cell lines. In fact, many cytotoxic compounds that are assayed on chemoresistant cancer cells are rejected by these cancer cells thanks to various efflux pumps that form the so-called multidrug-resistant phenotype [42]. Large numbers of cytotoxic compounds are already routinely used to combat chemosensitive cancers. However, when these chemosensitive cancers are chronically treated with cytotoxic and/or pro-apoptotic drugs, they develop chemoresistance, given that the MDR phenotype is very efficient at inhibiting the cytotoxic activity of a large set of compounds of interest [42]. Thus, a selective (normal/cancer cells) cytotoxic compound must be assayed in MDR cancer cell lines in addition to conventional cancer cell lines. If the compound of interest is not active against MDR cancer cells and is cytotoxic against conventional cancer cell lines, it cannot be considered as a potential anticancer agent, even though it is selective between normal and cancer cells. For example, some marine-derived fungal metabolites with pro-apoptotic activity are active against MDR cancer cells. These compounds include, for example, bostrycin (**20**), SZ-685C (**21**) and 1403P-3 (**22**) [80,82]. However, it is not possible to know if these compounds are actually potential anticancer agents or simply highly poisonous compounds. Bostrycin derivatives also display cytotoxic activity against normal cells [77]. The cytotoxic effects of pro-apoptotic compounds against MDR cancer cells must therefore also be assayed in normal cells. Finally, compounds that induce cytotoxicity in highly apoptosis-sensitive cancer cells through pro-apoptotic insults are actually no longer needed for the reasons explained in the previous section.

## 5. Conclusions

The burden of cancer continues to increase, leading to pronounced morbidity and mortality in both developed and developing countries. Despite the efforts of academic institutions and pharmaceutical companies, the chemotherapeutic arsenal has remained limited, primarily due to the emergence of drug-resistant cancer types. Both terrestrial and marine natural sources have provided several lead compounds that have led to the development of the majority of the anticancer drugs currently used in therapeutics.

Unlike marine macroorganisms, such as sponges and other sessile invertebrates, which have been extensively investigated, marine-derived fungi had been neglected until recently as a source of lead structures that could increase the clinical pipeline in chemotherapy research. Even so, growing numbers of marine-derived fungal secondary metabolites with interesting pharmacological activities that are considered valuable for the development of new chemotherapeutic agents have been reported over the last three decades. In this review, we have highlighted several marine-derived fungal metabolites that can modulate the activity of several key enzymes involved in tumor growth and metastasis, instead of having a limited pro-apoptotic effect. Because of their relevant and unconventional anticancer molecular mechanisms as well as their activity against MDR cancer cells, these metabolites are undoubtedly attractive as lead structures that should undergo further investigation, namely for the synthesis of analogs that could increase their selectivity and reduce their toxicity. We thus believe that a given compound of interest that displays *in vitro* cytotoxic effects against cancer cells can be claimed as a potential anticancer agent if and only if it is selective towards normal cells and MDR cancer cells. As explained previously, this cytotoxic compound of interest should also induce non-apoptotic cell death.

However, despite the identification of these promising candidates for pre-clinical development, it is expected that several additional lead structures will be discovered in the near future. The distinct biosynthetic capabilities of the marine-derived fungi as promoted by a distinct evolutionary pressure should be explored through a multidisciplinary approach in a way that increases the hit rate of novel lead structures with the potential to develop new anticancer drugs. Additional attempts should be undertaken to increase the contribution of obligate marine fungi as sources of new chemotherapeutic agents, as well as the systematic investigation of new marine fungal strains for which their biosynthetic potential has not yet been explored. An increased rate of discovery of novel lead compounds from marine fungi can also be achieved through new culturing techniques able to activate silent biogenetic gene clusters, leading to the production of distinct and unique metabolites that are not expressed under standard laboratory culturing conditions. These approaches include co-cultivation with either other fungal strains or bacteria, stress-inducing culturing methods and the OSMAC (One Strain Many Compounds) strategy.

The recent dereplication of several metabolites with relevant anticancer properties originally reported from marine macroorganisms, the possibility of culturing marine-derived fungi through cheap fermentation techniques, and the untapped number of new marine-derived fungal species that have yet to be discovered clearly classify these microorganisms as one of the most promising sources for the discovery of new anticancer lead structures.

The fact that there is still no single marine-derived fungal anticancer drug currently available on the market reflects the recent history of marine-derived fungi investigation for the discovery of new bioactive metabolites, as demonstrated in this review by their chemical diversity and promising anticancer properties. Thus, it would not be surprising if the current chemotherapeutic clinical pipeline were fed with marine-derived fungal agents in the near future.
